# Domestication has altered the ABA and gibberellin profiles in developing pea seeds

**DOI:** 10.1007/s00425-023-04184-2

**Published:** 2023-06-23

**Authors:** Jana Balarynová, Barbora Klčová, Danuše Tarkowská, Veronika Turečková, Oldřich Trněný, Martina Špundová, Sergio Ochatt, Petr Smýkal

**Affiliations:** 1 Department of Botany, Faculty of Science, Palacky University, 783 71 Olomouc, Czech Republic; 2 Laboratory of Growth Regulators, Palacky University and Institute of Experimental Botany, Czech Academy of Sciences, 783 71 Olomouc, Czech Republic; 3 Agriculture Research Ltd., 664 41 Troubsko, Czech Republic; 4 Department of Biophysics, Faculty of Science, Palacky University, 783 71 Olomouc, Czech Republic; 5 Agroécologie, InstitutAgro Dijon, INRAE, Univ. Bourgogne, Univ. Bourgogne Franche-Comté, 21000 Dijon, France

**Keywords:** Desiccation, Legume, Maturation, Phytohormones, Pigmentation, Seed-coat

## Abstract

**Main conclusion:**

We showed that wild pea seeds contained a more diverse combination of bioactive GAs and had higher ABA content than domesticated peas.

**Abstract:**

Although the role of abscisic acid (ABA) and gibberellins (GAs) interplay has been extensively studied in *Arabidopsis* and cereals models, comparatively little is known about the effect of domestication on the level of phytohormones in legume seeds. In legumes, as in other crops, seed dormancy has been largely or entirely removed during domestication. In this study, we have measured the endogenous levels of ABA and GAs comparatively between wild and domesticated pea seeds during their development. We have shown that wild seeds contained more ABA than domesticated ones, which could be important for preparing the seeds for the period of dormancy. ABA was catabolised particularly by an 8´-hydroxylation pathway, and dihydrophaseic acid was the main catabolite in seed coats as well as embryos. Besides, the seed coats of wild and pigmented cultivated genotypes were characterised by a broader spectrum of bioactive GAs compared to non-pigmented domesticated seeds. GAs in both seed coat and embryo were synthesized mainly by a 13-hydroxylation pathway, with GA_29_ being the most abundant in the seed coat and GA_20_ in the embryos. Measuring seed water content and water loss indicated domesticated pea seeds´ desiccation was slower than that of wild pea seeds. Altogether, we showed that pea domestication led to a change in bioactive GA composition and a lower ABA content during seed development.

**Supplementary Information:**

The online version contains supplementary material available at 10.1007/s00425-023-04184-2.

## Introduction

The seed consists of an embryo and an endosperm (resulting from double fertilization), which are enclosed in a maternally derived seed coat. In legumes, the seed coat and endosperm develop first, followed by the development of embryo (Weber et al. 2005). The endosperm is present only during early seed development and provides nutrients for the developing embryo. However, by approximately 17 days after pollination (DAP), the expanding embryo consumes the endosperm, and most of the seed comprises the growing embryo (Ribalta et al. 2017; Zablatzká et al. 2021). Nevertheless, to develop successfully, these three components must communicate with each other to coordinate their growth (Ochatt and Abirached-Darmency 2019). Growth is the result of a balance between many promoting and inhibiting factors, including plant hormones.

The seed development comprises three phases: histodifferentiation, maturation and desiccation, interlaced with two lag phases (Hedley and Ambrose 1980). During histodifferentiation, the embryo is produced through cell division and differentiation. After these events, cell expansion and deposition of stored reserves take place (maturation). Seed maturation is a physiological process accompanied by changes in levels of certain plant hormones, mainly gibberellins (GAs) and abscisic acid (ABA). Early embryo growth is mainly maternally controlled, and the transition into maturation indicates a switch to filial control. Finally, seed development terminates during maturation, and the seed enters a quiescence associated with a rapid decline in seed water content (Weber et al. 2005).

In legume seeds, physical dormancy develops during the later period of seed maturation. Physical dormancy (hardseededness) is characteristic of an impermeable seed coat, which does not allow water and gases to enter the seed. The mechanism underlying this phenomenon is not yet fully explained. However, it was shown that not only the seed coat thickness but also seed coat texture and biochemical and chemical composition are crucial components of this complex seed trait (Hradilová et al. 2017; Janská et al. 2019; Zablatzká et al. 2021). Unlike physiological dormancy, physical dormancy is not based on ABA and GAs balance. However, the model legume *Medicago truncatula* exhibits both physical and physiological dormancy (Ochatt and Abirached-Darmency 2019).

ABA is a sesquiterpene influencing many aspects of the plant lifecycle, including seed morphogenesis as well as germination. In the seed, the ABA hormone level results from transportation from the mother plant through the phloem and synthesis in the seed itself (Ali et al. 2022). Karssen et al. (1983) showed that ABA is synthesized especially during seed maturation, first in the seed coat and then at lower levels in the embryo and endosperm. The level of ABA decreases during desiccation and becomes relatively low in dry mature seeds.

ABA action in the seed has been shown to be the result of ABA synthesis, catabolism, transport and sensing (Ali et al. 2022). In plants, ABA is synthesized from a C_40_ carotenoid precursor via oxidative cleavage. The first oxygenated carotenoid precursor, zeaxanthin, is converted by zeaxanthin epoxidase (ZEP) into all-*trans*-violaxanthin and then to *trans*-neoxanthin, which are isomerized into the required *cis*-forms (North et al. 2007). The subsequent reaction leading to the next ABA precursor, xanthoxin (C_15_), is catalyzed by 9-*cis*-epoxycarotenoid dioxygenases (NCEDs), which are key regulators of ABA synthesis. Xanthoxin is then converted to abscisic aldehyde by a short-chain dehydrogenase/reductase (SDR1), and finally, the abscisic aldehyde is oxidized to ABA by abscisic aldehyde oxidase (AO) (Nambara and Marion-Poll 2003). Inactivation of ABA can be achieved by its oxidation at the C-7′, 8′ or C-9′ positions (Schwartz and Zeevaart 2010). The hydroxylation at the C-8′ position produces unstable 8′-hydroxy-ABA (8′-OH-ABA) that isomerizes spontaneously to phaseic acid (PA) and can be further reduced to dihydrophaseic acid (DPA). Hydroxylation at the 7′ and 9′ positions gives 7′-hydroxy-ABA (7′-OH-ABA) and 9′-hydroxy-ABA (9′-OH-ABA), respectively. 9′-OH-ABA cyclized to neophaseic acid (neoPA) (Zhou et al. 2004). ABA may also be inactivated by conjugation to glucose, producing a stored/transport form, the ABA glycosyl ester (ABA-GE) (Schwartz and Zeevaart 2010). ABA-GE can be converted back to free ABA by *β*-glucosidases (BGs) (Lee et al. 2006).

Both ABA and its cross-talk with other hormones and signalling molecules are crucial for seed development (Ali et al. 2022). GAs are known especially as growth-promoting plant hormones. Moreover, it was shown that bioactive GAs act as key mediators in growth responses to environmental cues (for example, light and temperature).

GAs are a family of diterpenoid carboxylic acids consisting of either 19 or 20 carbon atoms (Tarkowská and Strnad 2018). Among more than 130 known GAs, only a few are endogenous bioactive substances that control a wide range of plant growth and developmental processes, including seed germination. The major biologically active GAs are GA_1_, GA_3_, GA_4_, GA_5_, GA_6_ and GA_7_ (Yamaguchi 2008). The other GAs are either their inactive biosynthetic precursors or catabolites. Thus, the concentration of bioactive GAs is determined by the balance in their de novo biosynthesis and deactivation. In higher plants, GA_12_ is a precursor for all GAs which is produced by the oxidation of GA_12_-aldehyde by the enzyme ent-kaurenoic acid oxidase (KAO) (Hedden and Thomas 2012). There are two biosynthetic pathways leading to bioactive GAs: the 13-hydroxylation pathway (leading to the production of GA_1_ and GA_3_) and non-13-hydroxylation (leading to the production of GA_4_ and GA_7_) (Reinecke et al. 2013). The enzymes GA 20-oxidase and GA 3-oxidase are responsible for subsequent conversions of GA precursors and the production of bioactive GAs, respectively. The maintenance of the level of bioactive GAs is regulated by the action of GA 2-oxidase (Hedden and Thomas 2012).

Although the phytohormones stand behind the semi-dwarf varieties developed during the Green Revolution, their role in the early stages of the domestication process has not been studied so far. Since secondary metabolites pathways have often been altered (Alseekh et al. 2021), it is highly likely that phytohormone levels were also changed. Domestication affected many aspects of the plant lifecycle (Smýkal et al. 2018), including seed development. Particularly seed dormancy has been largely or entirely removed to allow the crop's rapid establishment. This is also the case with legumes (Smýkal et al. 2014), but its effect on the hormone profiles of developing seeds of wild and cultivated pea (legume) genotypes remains unknown. The aim of the present study was to investigate the role of ABA and GAs in embryo and seed coat development in wild and domesticated pea seeds. This might shed new light on understanding the differences in seed development in these accessions and provide a picture of the dynamics of pea seed development.

## Materials and methods

### Plant material

In these experiments, wild *Pisum elatius* M. Bieb. (JI1794, Israel origin), cultivated *Pisum sativum* L. (JI92, Afghan landrace) acquired from John Innes Pisum Collection (Norwich, UK), and the cultivated *P. sativum* cv. Cameor from INRAe France, used for the pea reference genome (Kreplak et al. 2019), were used. In addition, for seed water content and water loss measurements, wild JI64 pea (Turkey origin) was used instead of JI1794. Plants were cultivated and sampled as described in Zablatzká et al. (2021). The embryo and seed coat samples were collected at four time points: 13, 17, 23 (mid-development) and 28 DAP (mature seed), which were marked as developmental stages 1, 2, 3 and 4, respectively. At developmental stage 1, embryos of JI92 and JI1794 were not available in sufficient amounts and were thus not analyzed. Respective developmental stages were collected on several different days (i.e., the biological replicates). The seed coats and embryos of the same age (i.e., at the same developmental stage) but collected on different days were combined into one sample to cover the biological variation between different individuals and days of collection. Then, the sample was divided into three aliquots (replicates) for measurements.

### Isolation and analysis of ABA and its metabolites

Samples were extracted, purified and analysed according to a method described in Turečková et al. (2009). Briefly, 20 mg of plant tissue per sample was homogenized using a bead mill (27 Hz, 10 min, 4 °C; MixerMill, Retsch GmbH, Haan, Germany) and extracted in 1 ml of ice-cold methanol/water/acetic acid (10/89/1, by vol.) and internal standard mixtures, containing (-)-7′,7′,7′-^2^H_3_-phaseic acid; (-)-7′,7′,7′-^2^H_3_-dihydrophaseic acid; (-)-8′,8′,8′-^2^H_3_-neophaseic acid; ( +)-4,5,8′,8′,8′-^2^H_5_-ABA-GE; (-)-5,8′,8′,8′-^2^H_4_-7′-OH-ABA (National Research Council, Saskatoon, Canada) and (+)-3′,5′,5′,7′,7′,7′-^2^H_6_-ABA (OlChemIm, Olomouc, Czech Republic). After 1 h of shaking in the dark at 4 °C, the homogenates were centrifuged (20 000 g, 10 min, 4 °C), and the pellets were then re-extracted in 0.5 ml extraction solvent for 30 min. The combined extracts were purified by solid-phase extraction on Oasis^®^ HLB cartridges (60 mg, 3 ml, Waters, Milford, MA, USA), then evaporated to dryness in a Speed-Vac (UniEquip, Planegg, Germany) and finally analysed by UHPLC-ESI(-)-MS/MS (Waters, Manchester, UK).

### Isolation and analysis of gibberellins

The sample preparation and analysis of GAs were performed according to the method described in Urbanová et al. (2013) with some modifications. Briefly, tissue samples of about 5 mg (fresh weight, FW) were ground to a fine consistency using 2.7-mm zirconium oxide beads (Retsch GmbH) and a MM 400 vibration mill (Retsch GmbH) at a frequency of 27 Hz for 3 min with 1 ml of ice-cold 80% acetonitrile containing 5% formic acid as extraction solution. The samples were then extracted overnight at 4 °C using a benchtop laboratory rotator Stuart SB3 (Bibby Scientific Ltd., Staffordshire, UK) after adding internal gibberellins standards ([^2^H_2_]GA_1_, [^2^H_2_]GA_3_, [^2^H_2_]GA_4_, [^2^H_2_]GA_8_, [^2^H_2_]GA_9_, [^2^H_2_]GA_19_, [^2^H_2_]GA_20_, [^2^H_2_]GA_24_, [^2^H_2_]GA_29_, [^2^H_2_]GA_34_, [^2^H_2_]GA_44_, [^2^H_2_]GA_51_ and [^2^H_2_]GA_53_) purchased from OlChemIm. The homogenates were centrifuged at 36 670 g and 4 °C for 10 min, with the corresponding supernatants further purified using mixed-mode SPE cartridges (Waters, Milford, MA, USA) and analyzed by ultra-high performance liquid chromatography-tandem mass spectrometry (UHPLC-MS/MS; Micromass, Manchester, UK). GAs were detected using a multiple-reaction monitoring mode of the transition of the ion [M–H]^−^ to the appropriate product ion. Masslynx 4.2 software (Waters, Milford, MA, USA) was used to analyze the data, and the standard isotope dilution method (Rittenberg and Foster 1940) was used to quantify the GAs levels.

### Measurement of seed water content and water loss

Pea seeds were collected at five different developmental stages (13, 17, 23, 28, and 33 DAP, labeled as 1–5). Water loss experiments were performed according to Ranathunge et al. (2010) with few modifications. At the beginning of the experiment, FW of each seed was determined. Ten seeds of each stage were placed on a plate (Suppl. Fig. S1) and put into a glass desiccator filled with 400 g of freshly dried silica gel. Relative humidity in the desiccator was 4.9% ± 4.4%, measured with a humidity logger (Comet System, Rožnov pod Radhoštěm, Czech Republic) during the whole experiment. Seeds were weighed every hour for 5 h. All measurements were averaged and converted into percentages of seed FW. After water loss analysis, all seeds were dried in an oven at 103 °C for 17 h and dry weight (DW) was measured. Seed water content was calculated as the difference between seed FW and DW. The water loss rate was calculated as an average water loss per hour in % of FW. Data were calculated from 2 to 3 runs (measured in 2020 and 2021), each composed of 20–40 replicates per developmental stage. Experiments were performed at room temperature (around 23 °C).

### RNA sequencing

Frozen seed coats and embryos collected at four developmental stages 13, 17, 23 and 28 DAP (labelled as 1, 2, 3 and 4, respectively) were ground to a fine powder with liquid nitrogen, and total RNA was isolated using PureLink^™^ Plant RNA Reagent (Thermo Fisher Scientific, Waltham, MA, USA). Residual DNA was removed by Baseline-ZERO DNase (Epicenter, Madison, WI, USA) treatment followed by phenol/chloroform extraction. The RNA integrity of the samples was checked with an Agilent 2100 Bioanalyzer (Agilent Technologies, Palo Alto, USA). RNA sequencing was performed using Illumina NovaSeq platform performed by Novogene Ltd. (Cambridge, UK). The bioinformatics analysis of RNA-sequencing data was done as described in Balarynová et al. (2022). The expression level was normalized as FPKM (Fragments Per Kilobase Million).

### Statistical analysis

Since the data were not normally distributed, a distribution-independent test was used. Statistical analysis was performed by the Kruskal–Wallis test with the following post hoc non-parametric multiple comparisons (Siegel and Castellan 1988) at a 0.05 significance level using R 4.0.2. (R Core Team 2020).

## Results

### ABA is detected especially in the embryo, and dihydrophaseic acid is its predominant degradation product in pea seeds

**Fig. 1:**
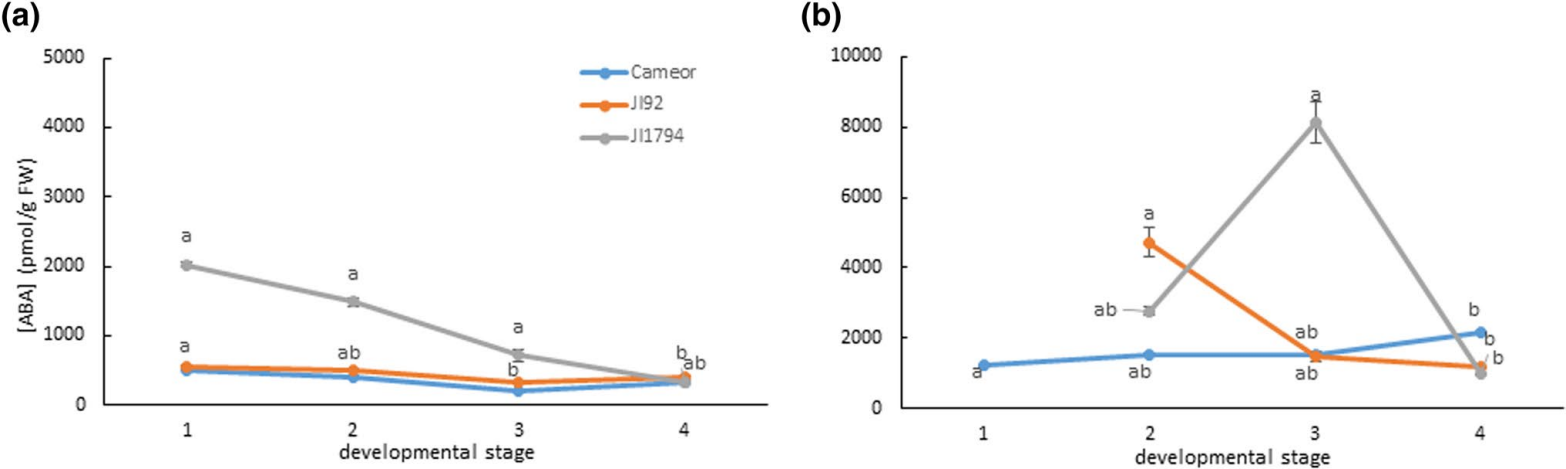
ABA levels in seed coats (**a**) and embryos (**b**) of cultivated (Cameor and JI92) and wild (JI1794) pea genotypes during the seed development. Data expressed means ± SD of three measurements. Different letters indicate significant differences (*P* = 0.05) between developmental stages of each genotype by the Kruskal–Wallis test with the following non-parametric multiple comparison test

The level of ABA was determined in the dissected seed coats and embryos of cultivated (Cameor, JI92) and wild (JI1794) pea genotypes, as shown in Fig. 1. The ABA contents in Cameor and JI92 seed coats were quite stable during development, while the ABA level in the seed coat of JI1794 (the wild pea) was higher than those of Cameor and JI92, and it decreased with the developmental stage. On the contrary, in the embryo, the ABA level increased with development, particularly in Cameor. Interestingly, the amount of ABA in JI1794 embryos decreased markedly after the 3rd developmental stage, while for JI92, the ABA content decreased constantly and progressively as the embryos matured. In our growth conditions, seeds were completely mature at around 28 DAP (4th developmental stage).

**Fig. 2:**
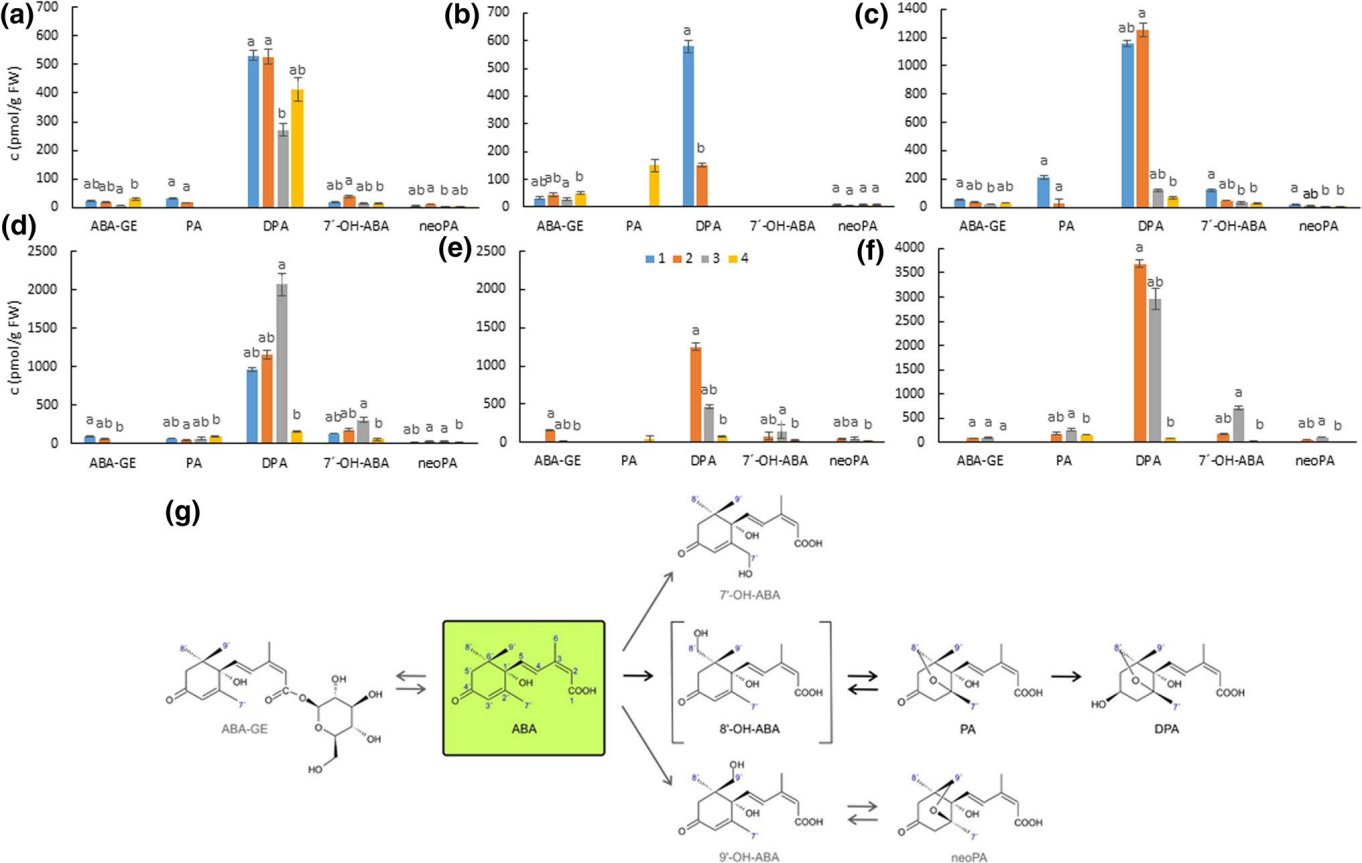
Quantification of ABA glycosyl ester (ABA-GE), phaseic acid (PA), dihydrophaseic acid (DPA), 7′-hydroxy-ABA (7′-OH-ABA) and neophaseic acid (neoPA) levels in the seed coats (**a**–**c**) and embryos (**d**–**f**) in the developing seeds of Cameor (**a**, **d**), JI92 (**b**, **e**) and JI1794 (**c**, **f**) peas. Data expressed mean ± SD of three measurements. Different letters indicate significant differences (*P* = 0.05) between developmental stages of particular metabolite by Kruskal–Wallis test with the following non-parametric multiple comparison test. The scheme of ABA inactivation (**g**)

To monitor the changes in ABA level in a broader context, the profiles of ABA metabolites were analysed during pea seed development as well (Fig. 2, Suppl. File S1). We found that DPA, which is produced from PA by 8'- hydroxylation pathway (Fig. 2g), was the major catabolic product in both seed coats and embryos, whereas neoPA, the product of the 9′-OH pathway, was the least abundant. Moreover, the levels of DPA correspond with levels of ABA in both tissues of all genotypes (except seed coats of JI92, which did not contain DPA at the 3rd and 4th developmental stages). In seed coats, PA was detected in the 1st and 2nd developmental stages of Cameor and JI64, while in JI92 it was found only in the latter stage. Similarly, in embryos, PA was identified in all studied developmental stages of Cameor and JI64, while in JI92 it was found only in the last developmental stage. On the other hand, 7′-hydroxy-ABA (7′-OH-ABA) was not detected in JI92 seed coat.

### GA_1_ and the gibberellins of the 13-hydroxylation pathway prevailed in the developing pea seeds

As mentioned above, not only ABA but also GAs play an important role during seed development. For this reason, the level of bioactive GAs, their biosynthetic precursors and catabolites were determined in developing seed coats and embryos of both cultivated (Cameor, JI92) and wild (JI1794) peas.

**Fig. 3:**
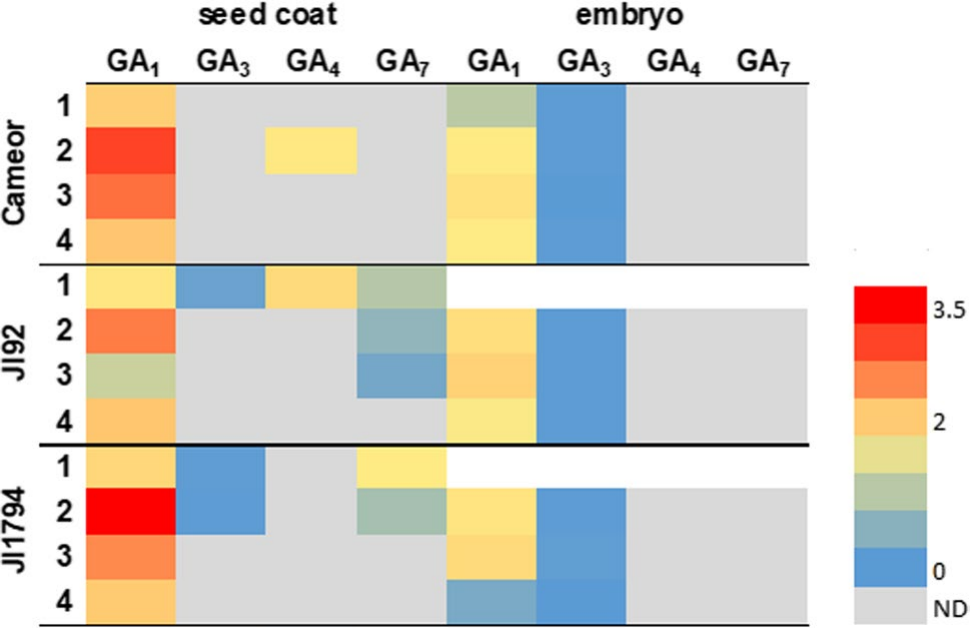
The bioactive GAs detected in developing seed coat and embryo of cultivated (Cameor, JI92) and wild (JI1794) pea genotypes at four developmental stages (1–4). The heatmap is based on average gibberellin content (pmol/g FW). *ND* not detected

In accordance with the literature (Garcia-Martinez et al. 1987; Graebe 1987), GA_1_ was found to be the main bioactive GA in the developing seeds of all three pea genotypes (Fig. 3). Besides GA_1_, also other bioactive GAs such as GA_3_ (from the 13-hydroxylation pathway), GA_4_ and GA_7_ (from the non-13-hydroxylation pathway) were detected (Fig. 3), especially in the seed coat. Interestingly, the seed coat of the pigmented pea genotypes (JI92 and JI1794) contained a more diverse combination of bioactive GAs than the seed coat of the non-pigmented cultivated Cameor seeds. Moreover, the seed coats produced more variable bioactive GAs than the corresponding embryos (Fig. 3).

**Fig. 4:**
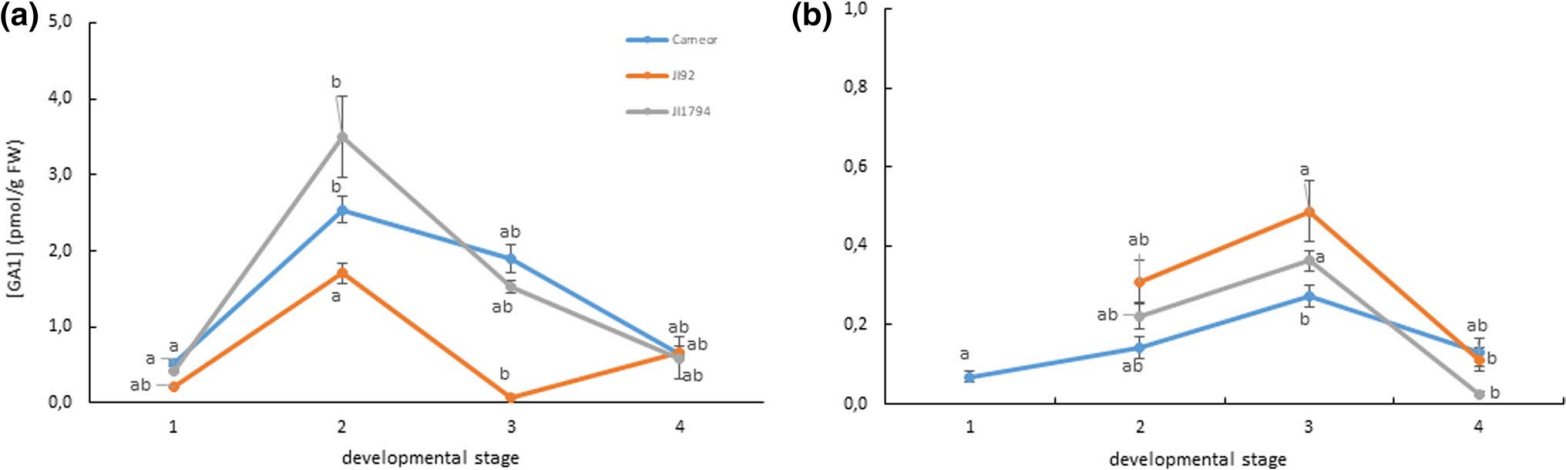
The level of GA_1_ during the development of cultivated (Cameor, JI92) and wild (JI1794) pea seed coat (**a**) and embryo (**b**). Data expressed mean ± SD of three independent measurements. Different letters indicate significant differences (*P* = 0.05) among developmental stages of each genotype by Kruskal–Wallis test with the following non-parametric multiple comparison test

Unlike ABA, the level of GA_1_ was found to be higher in the seed coat than in the embryos of all studied genotypes (except for embryos of JI92 in the 3rd developmental stage). The amount of GA_1_ in seed coat of all studied genotypes tended to be highest in the 2nd developmental stage, then it decreased when reaching later developmental stages. On the contrary, the level of GA_1_ in the embryos was the greatest in the 3rd developmental stage (Fig. 4).

**Fig. 5:**
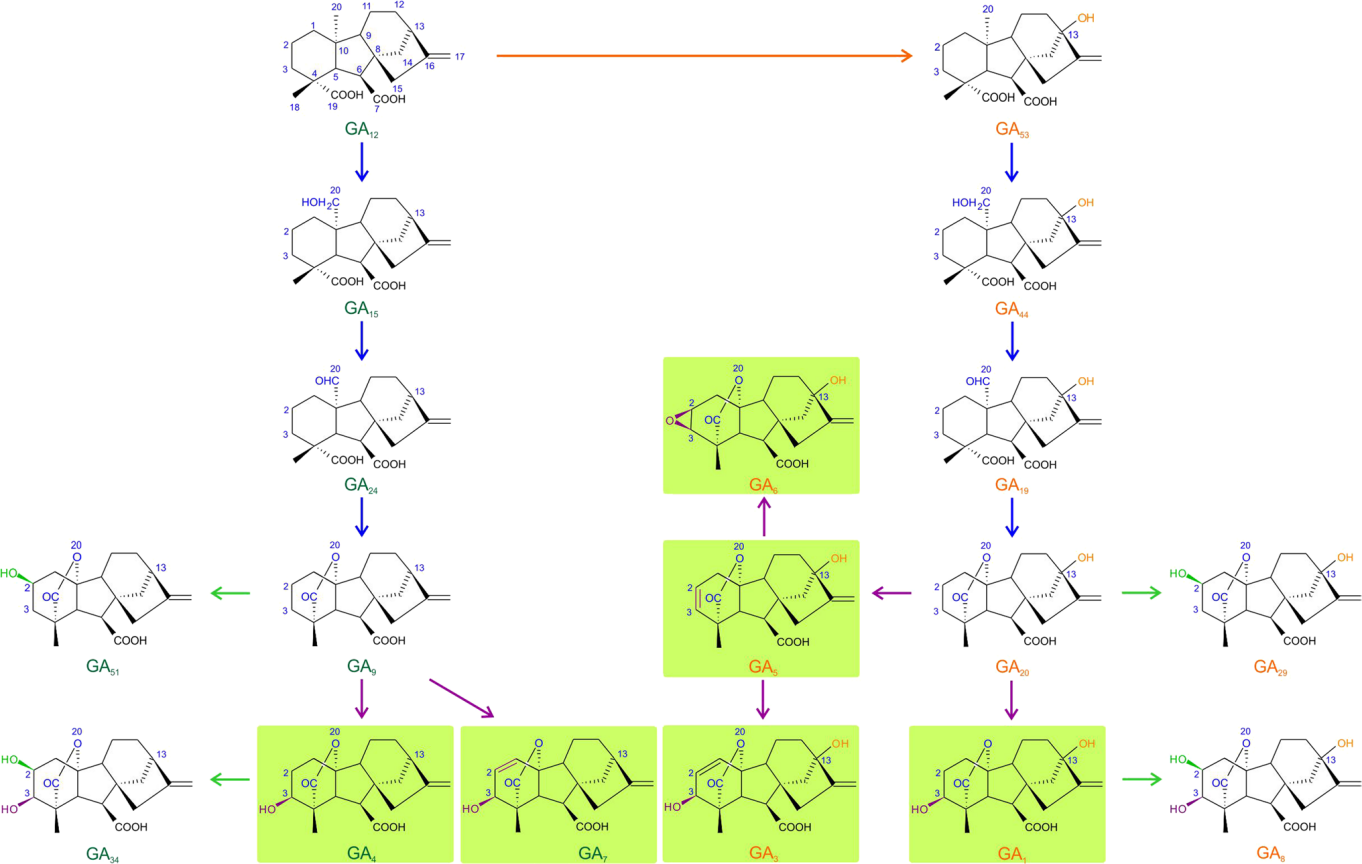
The simplified scheme of the non-13-hydroxylation (leading to the production of GA_4_ and GA_7_) and the 13-hydroxylation (leading to the production of GA_1_, GA_3_, GA_5_, GA_6_) gibberellin metabolic pathways. The bioactive GAs are in green rectangles. The arrows indicate enzymes responsible for GA precursor conversion, GA 20-oxidases (the blue arrows) and GA 3-oxidases (the violet arrows), and GA 2-oxidases (the green arrows) ensuring the conversion of bioactive GAs

**Fig. 6:**
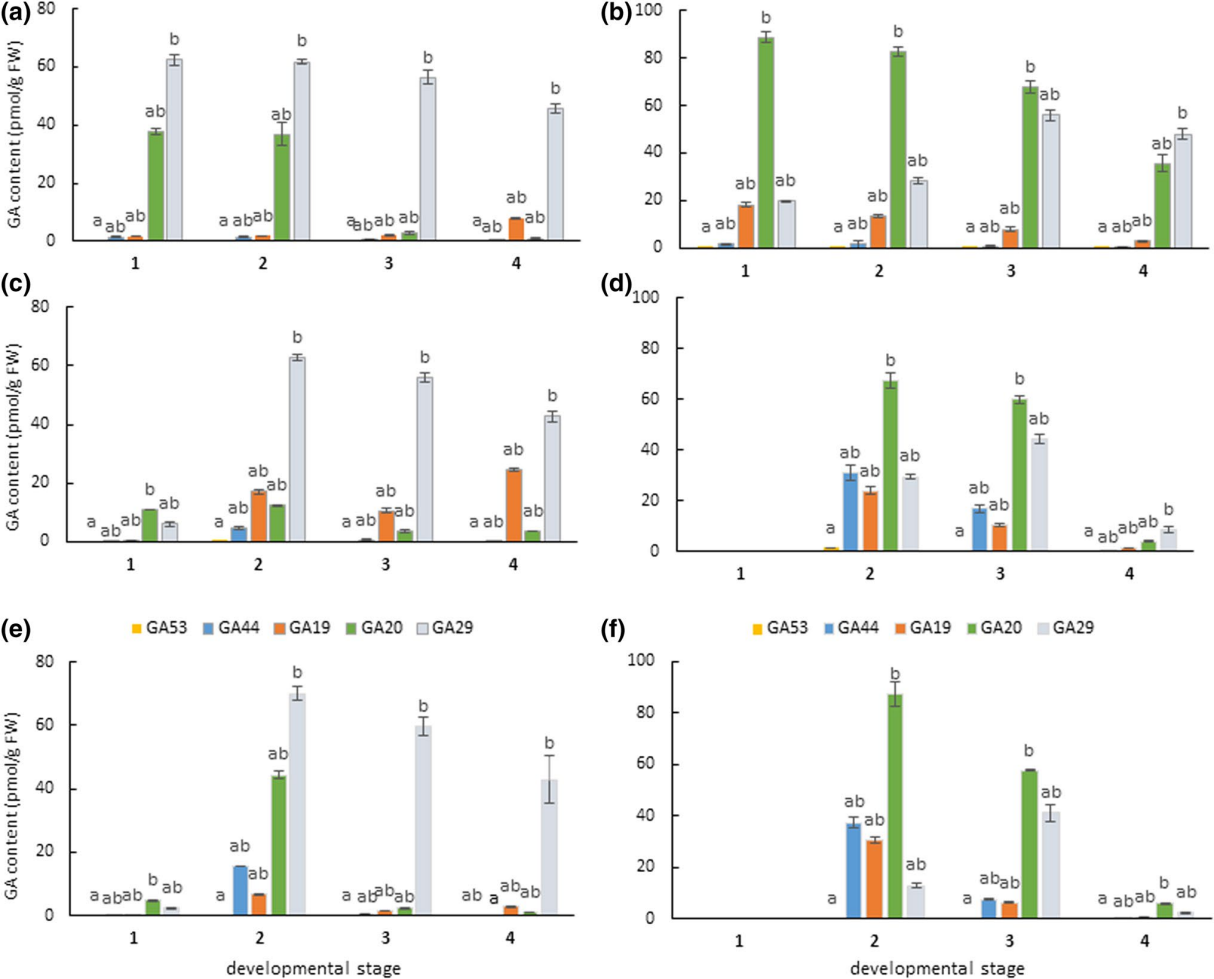
The level of GAs belonging to the 13-hydroxylation pathway in the developing seed coat (left panel) and embryo (right panel) of Cameor (**a**, **b**), JI92 (**c**, **d**) and JI1794 (**e**, **f**) pea genotypes. Data represent the mean ± SD of three independent measurements. Different letters indicate significant differences (*P* = 0.05) among various metabolites in each developmental stage by Kruskal–Wallis test with the following non-parametric multiple comparison test

GAs in both seed coat and embryo were biosynthesized mainly via the 13-hydroxylation pathway (Fig. 5). In our pea samples, GA_1_ biosynthetic precursors GA_53_, GA_44_, GA_19_, GA_20_, as well as GA_20_ degradation product GA_29_ were found (Fig. 6). Notably, GA_29_ was the most abundant gibberellin in the seed coat of all three studied pea genotypes, while GA_20_ was the major gibberellin detected in the embryos of both cultivated and wild peas. In contrast to Nadeau et al. (2011), GA_8_, the main degradation product of GA_1_, was detected only occasionally (its internal standards were recovered in all samples) and at a level close to the limit of detection of the method (data not shown).

**Fig. 7:**
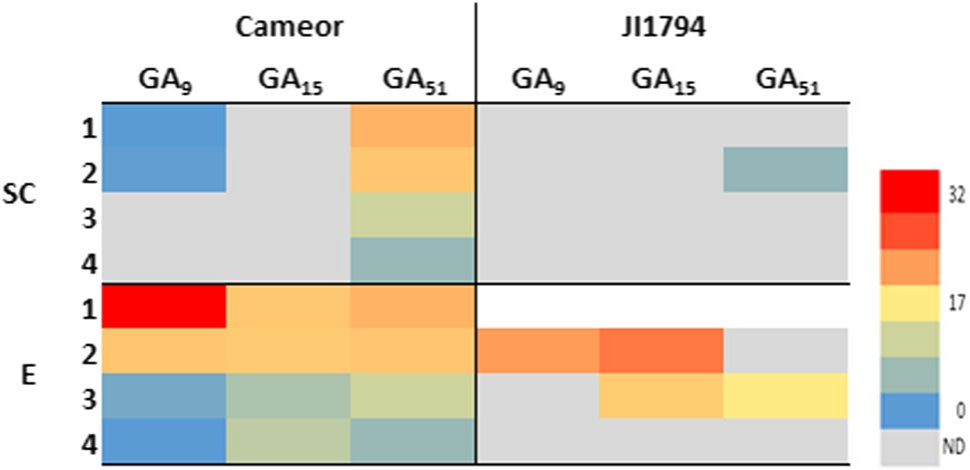
The precursors formed via the 13-non-hydroxylation pathway and detected in the seed coats (SC) and embryos (E) of cultivated (Cameor) and wild (JI1794) pea seeds at four developmental stages (1–4). The heatmap is based on average gibberellin content (pmol/g FW). The precursors of the 13-non-hydroxylation pathway were not detected in JI92 seed samples. *ND* not detected

The precursors belonging to 13-non-OH pathway (Fig. 5), GA_15_, GA_9_ and its degradation product GA_51_, were detected in both seed coats and embryos of Cameor and JI1794 (Fig. 7). However, they were not found in samples of JI92. Interestingly, GAs formed in this pathway were more abundant in the embryos compared to the seed coats. Moreover, they were found especially in Cameor genotype. The occurrence of 13-non-OH precursors significantly decreased in both genotypes along the development. Noteworthy, gibberellins GA_12_ and GA_24_ were not found in any tissue of all genotypes tested.

### Seed desiccation of domesticated pea is slower than those of landrace and wild pea

**Fig. 8:**
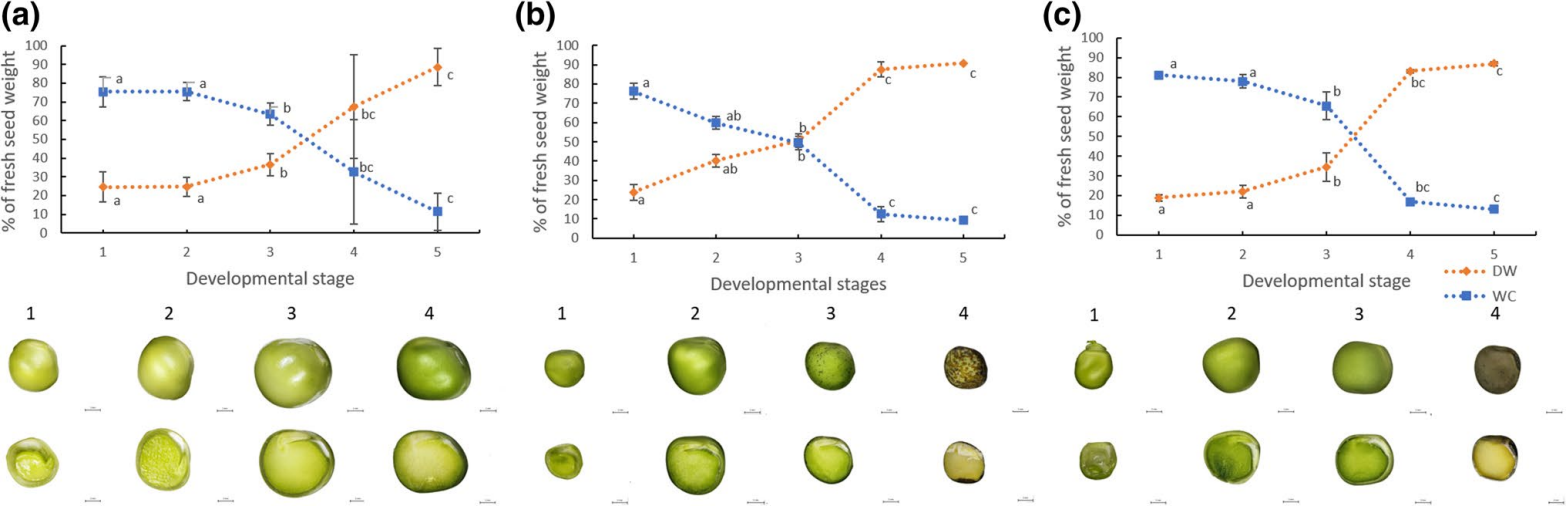
Maturation profiles of Cameor (**a**), JI92 (**b**), and JI64 (**c**) pea seed. Changes in seed water content (WC) and dry weight (DW) in cultivated (Cameor, JI92) and wild JI64 pea seed during development. The graphs show the percentage of WC and seed DW calculated from 2 to 3 experiments (± SD, *n* = 20–40 seeds). Different letters indicate significant differences (*P* = 0.05) by Kruskal–Wallis test with the following non-parametric multiple comparison test. Bars = 2 mm

The water content of seeds declined with the developmental stage (Fig. 8). At early stage 1, the water content of seeds was around 75–81% of seed FW, with the highest value for JI64. Seeds of wild pea genotype JI64 had the highest water content decline between stages 3 and 4, at around 47%, compared to domesticated and landrace genotypes. The greatest water content decline of landrace JI92 seeds, of around 40%, occurred between the 3rd and 4th developmental stages. This developmental time was associated with the desiccation (maturation) phase when the seed undergoes a fast drying process. In domesticated Cameor, the most rapid water content decrease was observed between the 3rd and 4th stages (about 28%), followed by about 24% between stages 4 and 5. Altogether, the domesticated pea desiccated more slowly and smoothly than primitive landrace JI92 and wild JI64. On the other hand, the desiccation profiles of JI92 and JI64 were characterized by a sudden change between the 3rd and 4th developmental stages.

**Fig. 9:**
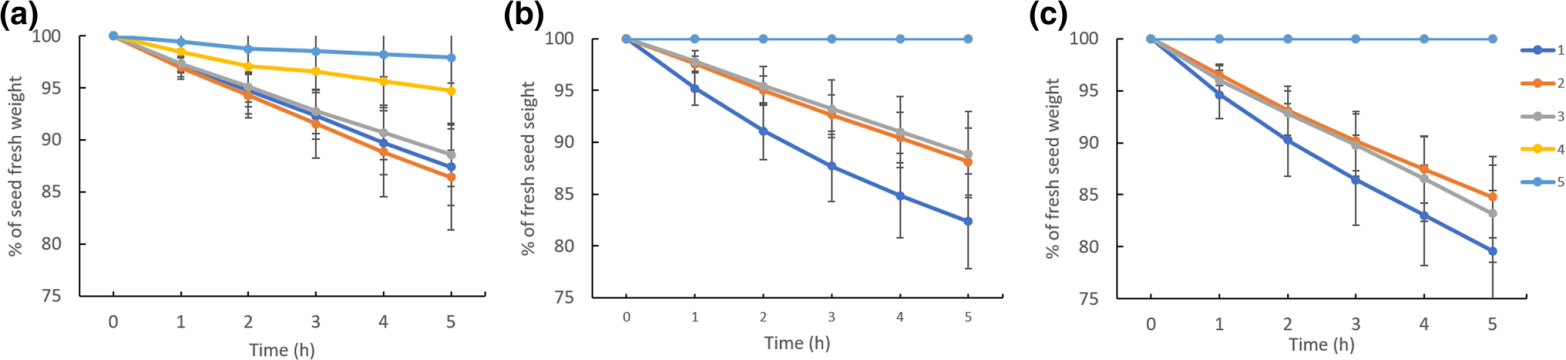
Water loss during seed development of Cameor (**a**), JI92 (**b**) and JI64 (**c**) pea genotypes. Stage 4 is not visible in **b** and **c** because it is hidden under the line presenting stage 5. Means ± SD calculated from two to three experiments (*n* = 20–40 seeds) are presented. The results of the statistical analysis are shown in Suppl. Fig. S3

Besides seed maturation profiles, the water loss rate was measured (Fig. 9). In Cameor (the domesticated pea), the water loss kinetics was similar in the first three developmental stages, then it declined in the 4th and 5th stages, without reaching zero (Fig. 9a, Table 1). Landrace JI92 lost the most significant amount of water in early stage 1 (about 3.5% of FW/hour), whereas in stages 2 and 3, water loss was around 2.3% of FW/h. In JI92, there was no measurable water loss in stages 4 and 5, as the seed water content was already minimal at these stages. (Fig. 9b, Table 1). In wild JI64, the most extensive water loss was detected in the first developmental stage (4.1% FW/h), followed by a decline in the 2nd and 3rd stages (3.0 and 3.4% of FW/h). Similarly to JI92 seeds, developmental stages 4 and 5 of wild JI64 showed no water loss (Fig. 9c, Table 1). Based on water content in these stages, JI92 and JI64 seeds might be considered as fully matured. Unlike landrace and wild peas, the seeds of domesticated pea still lost some water even at the point of expected seed maturity (Fig. 9a). JI92 water loss rate showed slower desiccation in stages 2 and 3, which could correspond to lower water content of JI92 seed compared to those of JI64 (Fig. 9, Table 1). Interestingly, the water content of Cameor and JI64 was similar in the first three developmental stages (Fig. 9), however, the water loss rate was higher in JI64 seeds (Table 1).

**Table 1:** The water loss rate in pea seeds expressed as the loss of seed mass during the time

Water loss rate (% of mass/hour)			
Developmental stage	Domesticated Cameor	Landrace JI92	Wild JI64
1	2.5 ± 0.2	3.5 ± 0.9	4.1 ± 0.8
2	2.7 ± 0.2	2.3 ± 0.1	3.0 ± 0.4
3	2.2 ± 0.2	2.2 ± 0.1	3.4 ± 0.4
4	1.0 ± 0.4	0.0 ± 0.0	0.0 ± 0.0
5	0.4 ± 0.2	0.0 ± 0.0	0.0 ± 0.0

Values were calculated from the slope of the line of fresh seed weight loss (water loss, Fig. 9) and represent the mean of 2–3 measurements (± SD, *n* = 20–40 seeds)

### Genes of ABA biosynthesis and GA metabolism are expressed mainly in developing seed coats

**Fig. 10:**
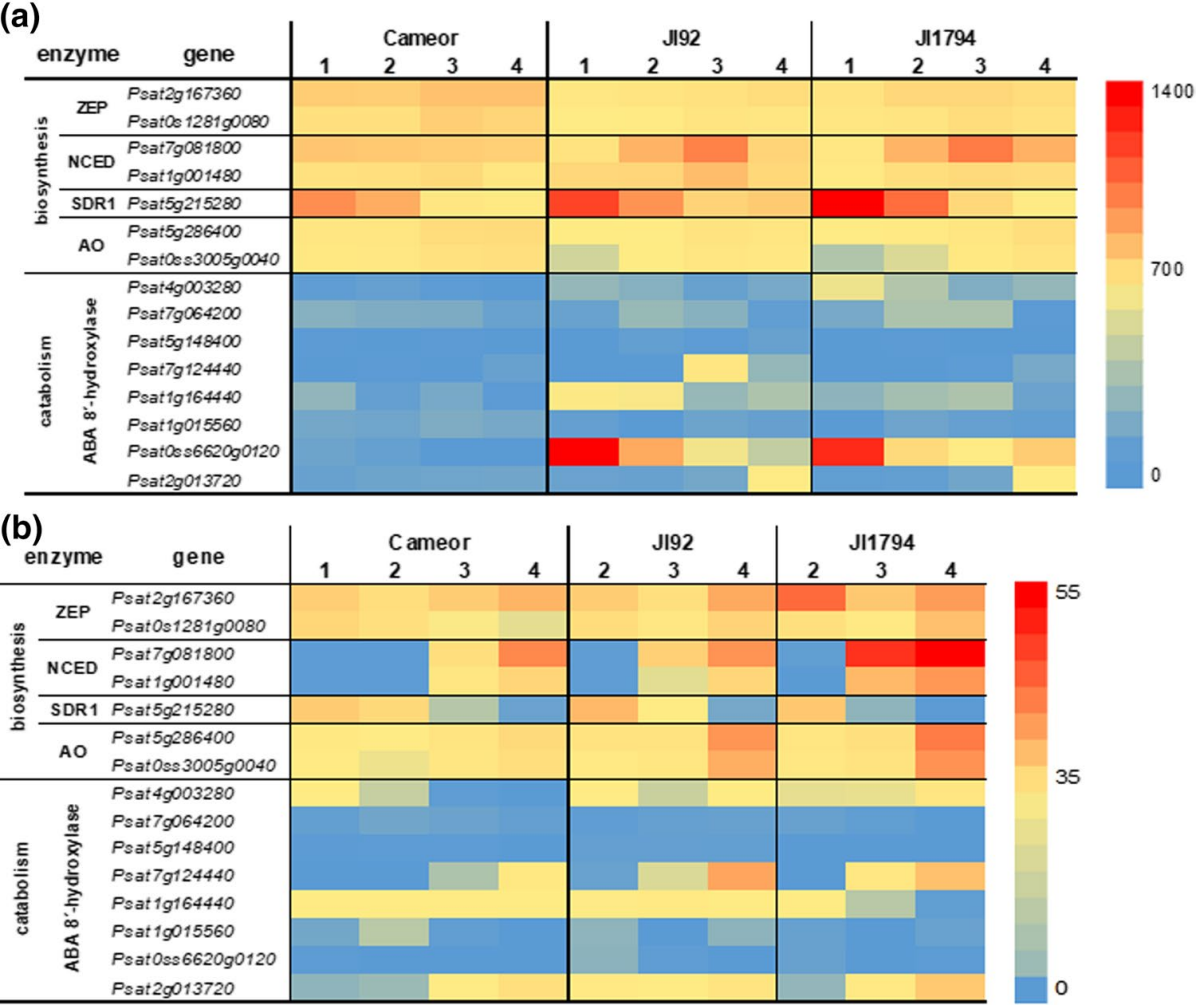
Heatmaps of genes involved in biosynthesis and catabolism of ABA in the seed coats (**a**) and embryos (**b**) of cultivated (Cameor, JI92) and wild (JI1794) peas. *ZEP* zeaxanthin epoxidase, *NCED* 9-*cis*-epoxycarotenoid dioxygenase, *SDR1* short-chain dehydrogenase reductase, *AO* abscisic aldehyde oxidase. The heatmap is based on average FPKM (Fragments Per Kilobase Million) values from RNA sequencing

To explore the temporal expression of ABA and GA metabolic genes, RNA sequencing data were searched for genes encoding enzymes of ABA and GA biosynthesis and catabolism. Heatmaps of transcription patterns of genes encoding key ABA metabolic enzymes (Fig. 10) showed the differences between tissues but also among genotypes. Generally, these genes were more transcribed in seed coats than in embryos. Genes encoding ABA biosynthetic enzymes tended to be expressed especially later in the seed development in both seed coats and embryos. Interestingly, in the Cameor seed coats *ZEP* and *AO* transcripts prevailed, whereas, in the seed coats of the pigmented genotypes (JI92 and JI1794), the expression of *NCED* (with a peak in the 3rd developmental stage) and *SDR1* (with a peak in the 1st developmental stage) genes were the strongest. *ABA 8*′*-hydroxylase* genes were expressed particularly at the first two developmental stages in the seed coats, particularly in the two pigmented genotypes, JI92 and JI1794. In the embryos, their expression augmented with the developmental stage.

**Fig. 11:**
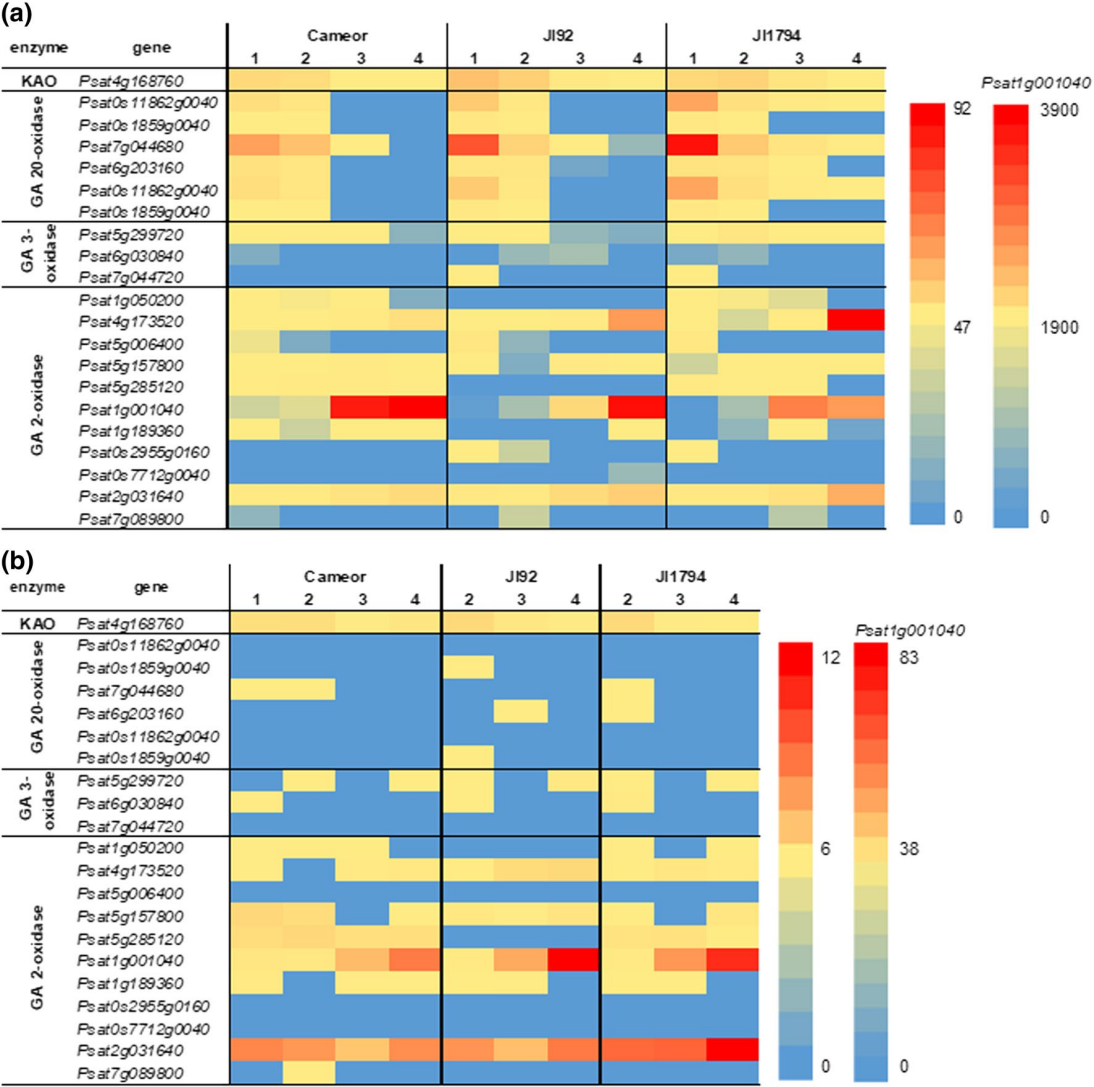
Heatmap analysis of genes involved in gibberellin metabolism in the seed coats (**a**) and embryos (**b**) of cultivated (Cameor, JI92) and wild (JI1794) peas. *KAO* *ent*-kaurenoic acid oxidase. The heatmap is based on average FPKM (Fragments Per Kilobase Million) values from RNA sequencing

Heatmaps of transcription patterns of genes encoding main GA metabolic enzymes (Fig. 11) showed that these genes were expressed mostly in the seed coats. In particular, *GA 20-oxidases* and *GA 3-oxidases* were transcribed mainly in the seed coats of all studied genotypes. In both the seed coats and embryos, the expression of genes encoding *KO*, *GA 20-oxidases* and *GA 3-oxidases*, which are involved in GA precursor formation and production of bioactive GAs, decreased with development. Their expression was higher in the seed coats of pigmented (JI92 and JI1794) genotypes compared to Cameor, while there was no difference among genotypes in the embryo samples. By contrast, the genes encoding enzymes deactivating bioactive GAs (*GA 2-oxidases*) were expressed predominantly later during seed development in both tissues similarly and in all three studied genotypes.

## Discussion

Seed germination is one of the key steps in a plant’s lifecycle. To germinate successfully, the seed development must be completed appropriately, and seed dormancy must be broken. The wild pea seeds will not germinate unless their seed coat is physically disrupted. The balance of phytohormones has a major influence on numerous morphological traits, including some of the agronomical relevance, such as seed dormancy, branching, tillering, and shoot and root architecture. Since seed dormancy is one of the key domestication traits (Hradilová et al. 2017; Smýkal et al. 2018), it is not surprising to see the alteration of hormone levels in crops compared to their wild progenitors (Ben-Abu and Itsko 2022). This has been nicely documented in the case of maize domestication (Dong et al. 2019).

Seed, and especially seed coat, structure (Zablatzká et al. 2021) and chemical composition (Janská et al. 2017; Hradilová et al. 2017) were studied in both wild and cultivated peas. However, the hormone levels were extensively monitored only in various pea cultivars (Ribalta et al. 2017, 2019). To the best of our knowledge, this study is the first one focused on ABA and GA changes between cultivated and wild peas during seed development. We focused on the later stages of seed development, from 13 to 28 DAP, in relation to our seed coat study (Balarynová et al. 2022). At the earliest developmental stage (13 DAP), the seed coat structure is already developed, both in wild and cultivated genotypes, and the embryo is embedded in the liquid endosperm (Zablatzká et al. 2021). After gradual endosperm consumption, the growing embryo comes into contact with the seed coat (by 17 DAP, in the second developmental stage). Endosperm depletion is connected with the expansion of parenchyma cells (the innermost layer of the seed coat), which forms nutritional tissue supporting the developing embryo with nutriments unloaded from the phloem (Nadeau et al. 2011). Accordingly, the peak in GA_1_ accumulation was found in the seed coat (Fig. 4) of both cultivated and wild peas by 17 DAP, which could be explained by the growth of the parenchyma layer of the seed coat. Subsequently, the developing embryo absorbs nutrients supplied by the seed coat, and the parenchyma is slowly crushed by the expanding cotyledons (Van Dongen et al. 2003). The further growth of the embryo corresponded to an increase in the level of GA_1_ (Fig. 4) in the third developmental stage (23 DAP); as the size of seeds increases, the process of desiccation begins, and the seed coat pigmentation (in JI92 and JI1794) starts to develop (Zablatzká et al. 2021).

Seed maturation is associated with reduced water content and changes in ABA content in the embryo. To survive the desiccation occurring during maturation, desiccation tolerance is acquired by an accumulation of antioxidants, various osmoprotectants, late embryogenesis abundant (LEA) proteins, and ABA plays a vital role in this process (Corbineau et al. 2000; Bewley et al. 2013). The level of ABA in the embryos reaches its peak just before the embryos start to desiccate (King 1976; Hsu 1979; Karssen et al. 1983). Afterward, the ABA level is quite low in mature seeds (Karssen et al. 1983). Our data showed that seeds of the semi-domesticated pea, landrace JI92, developed faster. The maturation began earlier than in seeds of wild or domesticated peas (Fig. 8). The peak of ABA level (Fig. 1), indicating the onset of maturation, was detected in the 2nd and 3rd developmental stages of JI92 and JI1794 embryos, respectively. The level of ABA in Cameor embryos started to increase after the 4th developmental stage (Fig. 1), indicating a slower development and late maturation. A similar trend was shown in experiments on the rate of water loss (Fig. 9, Table 1), which might hence be considered an indicator of seed maturation, the same as changes in ABA content. For some legumes, seed maturity was reported to be reached when water content drops to 55–60% of FW, when maximum dry weight is reached (Ellis et al. 1987). After finishing the seed-filling phase, seeds start desiccating, developing desiccation tolerance (Ellis et al. 1987). In pea, the desiccation tolerance is expected to evolve between 65 and 80% of water content before physiological maturity is acquired (Ellis et al. 1987; Ney et al. 1993). We showed that Cameor seeds typically exhibited a lower water content loss and a slower dry weight increase compared to JI92 (a primitive pea landrace) and wild pea seeds (Figs. 8 and 9, Table 1), which indicated the slower development of Cameor seeds. Moreover, the Cameor seeds showed a slower water loss rate than wild seeds during the first three developmental stages, although their water contents were quite similar. It was unclear why the seeds of domesticated pea lost water more slowly, perhaps this could be related to their lower water potential at the given stages, but this would need to be verified. Matthews (1973) suggested that an initial slow water content decrease helps to develop desiccation tolerance in pea seeds. According to our data, we can assume that maturity was acquired between 2nd and 3rd developmental stages in JI92 seeds, while in JI64 and Cameor seeds it tended to be achieved later, between 3rd and 4th developmental stages.

Comparative analysis of embryos and seed coats of wild and cultivated pumpkin (*Cucurbita maxima*), which has a combination of physical and physiological dormancy, like the model legume *M. truncatula* (Ochatt and Abirached-Darmency 2019), has revealed that while in the embryos, ABA concentrations were similar in both domesticated and wild subspecies, in seed coats, it was threefold higher in the wild subspecies (Martínez et al. 2018). Moreover, in this study with pumpkin, the naked embryos from the wild subspecies were far more responsive to ABA than those from the domesticated subspecies. These results indicate that dormancy in the wild pumpkin is imposed by the seed coat tissues and that this effect is mediated by their high ABA content and the sensitivity of the embryos to ABA (Martínez et al. 2018). Unlike pumpkin, ABA and its metabolites were more present in embryos than in seed coats of all pea genotypes studied (Figs. 1 and 2). Both tissues of the wild pea had the highest amount of ABA and its metabolites (Suppl. File S1) which could be explained by its great resilience and ability to survive in the changing natural localities. The high content of ABA is usually connected with dormancy and resistance to various biotic and abiotic challenges, as shown in pea (Ochatt 2015; Ribalta et al. 2019). It is known that ABA plays an important role in the initiation of dormancy in developing seeds (Feurtado and Kermode 2007). Although wild pea seed dormancy is primarily determined by the water-impermeable seed coat, and its acquisition in legumes is still poorly understood, the elevated ABA level during seed development might be important to prepare the embryo for survival in the dry dormant seed. Similarly to *Arabidopsis thaliana* seeds, ABA might be involved in the establishment of seed coat impermeability by regulating the synthesis of various compounds such as the hydroxylated fatty acids, phenolics, or pigments (Mendoza et al. 2005), which were shown to be more abundant in the seed coat of wild peas (Cechová et al. 2017; Janská et al. 2019; Krejčí et al. 2022). Pea seed coat showed abundant accumulation of phenolic compounds that, upon oxidation, may impact seed permeability as well as pigmentation, both typical in the seed coat of dormant peas (Balarynová et al. 2022; Krejčí et al. 2022).

Gene expression of ABA and GA metabolism genes has been studied during seed development and imbibition in rice, the species with physiological dormancy (Liu et al. 2014). A comparison of dormant and non-dormant seeds showed differences in the peak of *OsNCED* (ABA biosynthesis) between dormant and non-dormant genotypes. On the contrary, our data rather showed a difference between non-pigmented (Cameor, stable *NCED* gene expression) and pigmented genotypes (JI92 and JI1794, *NCED* gene expression peaked at 3rd developmental stage) instead, with no effect of dormancy (physical dormancy) (Fig. 10). This could indicate the relationship between seed coat pigmentation and ABA. Similarly, *ABA 8′-hydroxylase* genes expression showed similar trends (Fig. 10). On the other hand, *GA 20-oxidases* and *GA 3-oxidases* showed several peaks during the non-dormant rice seed development, whereas their expression in dormant seeds remained stable. This corresponds to a lower accumulation of active GAs in non-dormant genotype (Liu et al. 2014). Conversely, the main difference between studied genotypes in our study was not in the expression patterns of GA metabolism genes but in their levels of expression, which were higher in the pigmented genotypes (Fig. 11). Although the expression of GA and ABA-related genes was analyzed only in cultivated legumes, such as alfalfa (*Medicago sativa*) (Zhao et al. 2022), their expression patterns were in agreement with our data. Finally, we showed that both ABA and GA metabolic genes were expressed predominantly in the seed coat, indicating the crucial role of seed coat in governing ABA and GA hormonal levels in the developing pea seeds.

Based on seed coat pigmentation, the pea genotypes studied here can be divided into pigmented (JI92 and JI1794) and non-pigmented (Cameor) ones (Suppl. Fig. S2). Despite being pigmented, JI92 is a primitive domesticated landrace compared to JI1794, which belongs to wild pea genotypes. It has been shown that cultivated legume seeds contain fewer carotenoids, the precursors of ABA, than their wild counterparts (Frey et al. 2006; Fernández-Marín et al. 2014). Moreover, ABA is also able to promote or inhibit the biosynthesis of anthocyanin in fruits by cross-talking with other phytohormones including jasmonic acid, GAs, auxin and cytokinin (Xie et al. 2012; Jaakola 2013; An et al. 2018). ABA level and the expression of *NCED* were associated with pigment production (Jia et al. 2011; Karppinen et al. 2018; Li et al. 2019). Correspondingly, the expression of *NCED* was increased in the seed coat of pigmented peas (JI92 and JI1794) (Fig. 10). In peanut (*Arachis hypogaea*), an association not only between ABA signalling and anthocyanins but also proanthocyanidin (condensed tannins) content was suggested (Wan et al. 2016). The fact that proanthocyanidins stimulate ABA synthesis was also observed during the maturation and germination of *Arabidopsis* seeds (Jia et al. 2012).

On the other hand, it has been shown that proanthocyanidins can also act as GA antagonists in pea (Green and Corcoran 1975; Corcoran et al. 1972). In common bean (*Phaseolus vulgaris*) seeds, (+)- catechin (found mainly in seed coat, not in embryo) inhibits the conversion of GA_12_-aldehyde to GA_12_ (Kwak et al. 1988). In our samples, we detected GAs of the 13-non-hydroxylation pathway, especially in Cameor (Fig. 7), the domesticated genotype with the seed coat low in proanthocyanidins (Hradilová et al. 2017). Besides, GAs were shown to regulate anthocyanin biosynthesis (Loreti et al. 2008). Unlike ABA, the seed coat of the studied pea genotypes contained more bioactive GAs than the embryo (Figs. 3 and 4). Moreover, the seed coat of pigmented genotypes (primitive domesticated landrace JI92 and wild JI1794) contained a more diverse combination of bioactive GAs than Cameor (Fig. 3). The seed coat of JI92 and JI1794 contained GA_3_ and GA_7_, which were not detected in Cameor. Thus, we might expect that these GAs may play a role in the development of seed coat pigmentation.

Our data showed that GA_20_ and GA_29_ were the most abundant gibberellins detected in the embryos and seed coats (Fig. 6), respectively, of studied pea genotypes. This is in agreement with previous work on immature pea seeds (Sponsel 1983; Zhu et al. 1991). In seeds, GA_20_ is metabolised to GA_29_ in the embryos and then it is transported to the seed coats. In the seed coat, GA_29_ is metabolised to GA_29_-catabolite (Sponsel 1983). This could explain a decrease in GA_29_ content with development despite very high gene expression of *GA 2-oxidases* in the later stages (Fig. 11).

Alteration of flavonoid pigmentation during crop domestication has been widely reported. Particularly, a loss of pigmentation in the edible parts is one of the domestication symptoms (reviewed in Smýkal et al. 2018; Paauw et al. 2019; Alseekh et al. 2021). The discussion on whether this is the result of direct selection or of linkage of other important domestication genes is ongoing. The consumer preference for visual appearance likely drives acting selection. This also acted in the case of grain legumes, such as pea, chickpea, common bean and lentil (Balarynová et al. 2022).

Notably, Wang et al. (2018) identified a gene responsible for seed dormancy that has been subjected to parallel selection in multiple crops. This gene encodes stay-green G gene-affected seed dormancy in soybean through interactions with NCED3 and PSY and in turn, modulated ABA synthesis. The green soybean seed coat is governed by three classical stay-green loci with different mechanisms, among which the G locus specifically dominantly controls the green colour of the seed coat, whereas the other two loci affect other organs as well. Using transgenic and mutant lines, they have shown that in the mutant *g* lines, less ABA is produced, resulting in the weakening of dormancy and thus facilitating crop management for farmers, which probably led to the parallel selection of g genotypes in various crops. Interestingly, the region around the G locus exhibits selection signatures in soybean domestication. However, if the trait under selection is seed coat colour, it is then perplexing. All wild soybeans with black seeds contain the G allele conferring the green seed coat colour, considering green is invisible against black. It was hypothesized that the trait controlled by G under selection is the reduction of seed dormancy. Indeed, overexpressing the wild-type G allele strengthened seed dormancy. However, G corresponds to a different mechanism from both, and it is a new gene linked to physiological dormancy. Whether this is a case of pea seed dormancy remains to be shown. However, both comparative transcriptomics and genetic mapping did not show any direct involvement of such genes (Hradilová et al. 2017 and unpublished results).

Analysis of seed content of different hormones suggests that the hormonal balance between ABA, GAs, and auxins at crucial time points during this process might underlie seed development differences in these accessions and would thus illustrate the dynamics of pea seed development. Auxin acts upstream of GA during seed coat development (Figueiredo et al. 2016). Despite the fact that the role of hormones in regulating legume seed development is poorly described, it was shown that the embryos of pea seeds do not germinate until physiological maturity (around 18 DAP) is reached (Ribalta et al. 2017), except if cultured in vitro in the presence of exogenous growth regulators (Ribalta et al. 2019). In these studies, it was found that cultivated pea seeds had the highest ABA level after physiological maturity, which can be linked to the biosynthetic pathway for ABA and the positioning of carotenoid biosynthesis in it (Nambara and Marion-Poll 2003; North et al. 2007; Ali et al. 2022).

## Conclusion

In this study, we provide the first report of ABA and GAs profiling wild pea seeds during their development and compare them to domesticated peas. Despite the loss of seed dormancy in domesticated legumes, the mechanisms underlying physical dormancy in legumes are still poorly understood. Our data showed that wild pea seed coat and embryo were abundant in ABA and its metabolites, which might be associated with the preparation of its seeds for a period of dormancy and the development of seed coat pigmentation. The seed coats of pigmented seeds differed in the composition of bioactive GAs and were transcriptionally more active in the expression of ABA and GA metabolite genes, highlighting the importance of seed coat during seed development.

*Author contribution statement* JB, PS conceived, and JB coordinated the research. JB, BK, DT, and VT performed the experiments and JB, BK, DT, VT, and MŠ analysed data. OT contributed to RNA seq data analysis. The first draft of the manuscript was written by JB, SO, PS, all authors commented on previous versions of the manuscript and complemented it. All authors read and approved the final manuscript.

## Supplementary Information

Below is the link to the electronic supplementary material.

Supplementary file1 (DOCX 620 KB)

Suppl. File S1 Summary of statistical analysis of differences among genotypes. Different letters indicate significant difference (P = 0.05) by Kruskal–Wallis test with the following non-parametric multiple comparison test. Supplementary file2 (DOCX 43 KB)

## Data Availability

All data generated or analysed during this study are included in this published article and its supplementary information files.

## Abbreviations

DAP: Days after pollination
GAs: Gibberellins
NCED: 9-*cis*-Epoxycarotenoid dioxygenase

## References

[CR1] Ali F, Qanmber G, Li F, Wang Z (2022). Updated role of ABA in seed maturation, dormancy, and germination. J Adv Res.

[CR2] Alseekh S, Scossa F, Wen W, Luo J, Yan J, Beleggia R, Klee HJ, Huang S, Papa R, Fernie AR (2021). Domestication of crop metabolomes: desired and unintended consequences. Trends Plant Sci.

[CR3] An JP, Yao JF, Xu RR, You CX, Wang XF, Hao YJ (2018). Apple bZIP transcription factor MdbZIP44 regulates abscisic acid-promoted anthocyanin accumulation. Plant Cell Environ.

[CR4] Balarynová J, Klčová B, Sekaninová J, Kobrlová L, Cechová MZ, Krejčí P, Leonova T, Gorbach D, Ihling C, Smržová L, Trněný O, Frolov A, Bednář P, Smýkal P (2022). The loss of polyphenol oxidase function is associated with hilum pigmentation and has been selected during pea domestication. New Phytol.

[CR5] Ben-Abu Y, Itsko M (2022). Metabolome dynamics during wheat domestication. Sci Rep.

[CR6] Bewley JD, Bradford KJ, Hilhorst HWM, Nonogaki H, Bewley JD, Bradford KJ, Hilhorst HWM, Nonogaki H (2013). Development and aturation. Seeds: physiology of development, germination and dormancy.

[CR7] Cechová M, Válková M, Hradilová I, Janská A, Soukup A, Smýkal P, Bednář P (2017). Towards better understanding of pea seed dormancy using laser desorption/ionization mass spectrometry. Int J Mol Sci.

[CR8] Corbineau F, Picard M, Fougereux J, Ladonne F, Côme D (2000). Effects of dehydration conditions on desiccation tolerance of developing pea seeds as related to oligosaccharide content and cell membrane properties. Seed Sci Res.

[CR9] Corcoran MR, Geissman TA, Phinney BO (1972). Tannins as gibberellin antagonists. Plant Physiol.

[CR10] Dong Z, Xiao Y, Govindarajulu R, Feil R, Siddoway ML, Nielsen T, Lunn JE, Hawkins J, Whipple C, Chuck G (2019). The regulatory landscape of a core maize domestication module controlling bud dormancy and growth repression. Nat Commun.

[CR11] Ellis RH, Hong TD, Roberts EH (1987). The development of desiccation-tolerance and maximum seed quality during seed maturation in six grain legumes. Ann Bot.

[CR12] Fernández-Marín B, Milla R, Martín-Robles N, Arc E, Kranner I, Becerril JM, García-Plazaola JI (2014). Side-effects of domestication: cultivated legume seeds contain similar tocopherols and fatty acids but less carotenoids than their wild counterparts. BMC Plant Biol.

[CR13] Feurtado JA, Kermode AR, Bradford K, Nonogaki H (2007). A merging of paths: abscisic acid and hormonal cross-talk in the control of seed dormancy maintenance and alleviation. Annual plant reviews: seed development, dormancy and germination.

[CR14] Figueiredo DD, Batista RA, Roszak PJ, Hennig L, Köhler C (2016). Auxin production in the endosperm drives seed coat development in *Arabidopsis*. Elife.

[CR15] Frey A, Boutin J-P, Sotta B, Mercier R, Marion-Poll A (2006). Regulation of carotenoid and ABA accumulation during the development and germination of *Nicotiana plumbaginifolia* seeds. Planta.

[CR16] Garcia-Martinez JL, Sponsel VM, Gaskin P (1987). Gibberellins in developing fruits of *Pisum sativum* cv. Alaska:studies on their role in pod growth and seed development. Planta.

[CR17] Graebe JE (1987). Gibberellin biosynthesis and control. Annu Rev Plant Physiol.

[CR18] Green FB, Corcoran MR (1975). Inhibitory action of five tannins on growth induced by several gibberellins. Plant Physiol.

[CR19] Hedden P, Thomas SG (2012). Gibberellin biosynthesis and its regulation. Biochem J.

[CR20] Hedley CL, Ambrose MJ (1980). An analysis of seed development in *Pisum sativum* L. Ann Bot.

[CR21] Hradilová I, Trněný O, Válková M, Cechová M, Janská A, Prokešová L, Aamir K, Krezdorn N, Rotter B, Winter P, Varshney RK, Soukup A, Bednář P, Hanáček P, Smýkal P (2017). A combined comparative transcriptomic, metabolomic, and anatomical analyses of two key domestication traits: Pod dehiscence and seed dormancy in pea (*Pisum* sp.). Front Plant Sci.

[CR22] Hsu FC (1979). Abscisic acid accumulation in developing seeds of *Phaseolus vulgaris* L. Plant Physiol.

[CR23] Jaakola L (2013). New insights into the regulation of anthocyanin biosynthesis in fruits. Trends Plant Sci.

[CR24] Janská A, Pecková E, Sczepaniak B, Smýkal P, Soukup A (2019). The role of the testa during the establishment of physical dormancy in the pea seed. Ann Bot.

[CR25] Jia HF, Chai YM, Li CL, Lu D, Luo JJ, Qin L, Shen YY (2011). Abscisic acid plays an important role in the regulation of strawberry fruit ripening. Plant Physiol.

[CR26] Jia L, Wu Q, Ye N, Liu R, Shi L, Xu W, Zhi H, Rahman ANMRB, Xia Y, Zhang J (2012). Proanthocyanidins inhibit seed germination by maintaining a high level of abscisic acid in *Arabidopsis thaliana*. J Integ Plant Biol.

[CR27] Karppinen K, Tegelberg P, Häggman H, Jaakola L (2018). Abscisic acid regulates anthocyanin biosynthesis and gene expression associated with cell wall modification in ripening bilberry (*Vaccinium myrtillus* L.) fruits. Front Plant Sci.

[CR28] Karssen CM, Brinkhorst-van der Swan DLC, Breekland AE, Koornneef M (1983). Induction of dormancy during seed development by endogenous abscisic acid: studies on abscisic acid deficient genotypes of *Arabidopsis thaliana* (L.) Heynh. Planta.

[CR29] King RW (1976). Abscisic acid in developing wheat grains and its relationship to grain growth and maturation. Planta.

[CR30] Krejčí P, Cechová MZ, Nádvorníková J, Barták P, Kobrlová L, Balarynová J, Smýkal P, Bednář P (2022). Combination of electronically driven micromanipulation with laser desorption ionization mass spectrometry–The unique tool for analysis of seed coat layers and revealing the mystery of seed dormancy. Talanta.

[CR31] Kwak S-S, Kamiya Y, Sakurai A, Takahashi N (1988). Isolation of a gibberellin biosynthesis inhibitor from testas of *Phaseolus vulgaris* L. Agric Biol Chem.

[CR32] Lee KH, Piao HL, Kim HY, Choi SM, Jiang F, Hartung W, Hwang I, Kwak JM, Lee I-J, Hwang I (2006). Activation of glucosidase via stress-induced polymerization rapidly increases active pools of abscisic acid. Cell.

[CR33] Li G, Zhao J, Qin B, Yin Y, An W, Mu Z, Cao Y (2019). ABA mediates development-dependent anthocyanin biosynthesis and fruit coloration in *Lycium* plants. BMC Plant Biol.

[CR34] Liu Y, Fang J, Xu F, Chu J, Yan C, Schläppi MR, Wang Y, Chu C (2014). Expression patterns of ABA and GA metabolism genes and hormone levels during rice seed development and imbibition: a comparison of dormant and non-dormant rice cultivars. J Genet Genom.

[CR35] Loreti E, Povero G, Novi G, Solfanelli C, Alpi A, Perata P (2008). Gibberellins, jasmonate and abscisic acid modulate the sucrose-induced expression of anthocyanin biosynthetic genes in *Arabidopsis*. New Phytol.

[CR37] Martínez AB, Lema V, Capparelli A, Anido FL, Benech-Arnold R, Bartoli CG (2018). Differences in seed dormancy associated with the domestication of *Cucurbita maxima*: elucidation of some mechanisms behind this response. Seed Sci Res.

[CR38] Matthews S (1973). Changes in developing pea (*Pisum sativum*) seeds in relation to their ability to withstand desiccation. Ann Appl Biol.

[CR39] Mendoza MS, Dubreucq B, Miquel M, Caboche M, Lepiniec L (2005). LEAFY COTYLEDON 2 activation is sufficient to trigger the accumulation of oil and seed specific mRNAs in *Arabidopsis* leaves. FEBS Lett.

[CR40] Nadeau CD, Ozga JA, Kurepin LV, Jin A, Pharis RP, Reinecke DM (2011). Tissue-specific regulation of gibberellin biosynthesis in developing pea seeds. Plant Physiol.

[CR41] Nambara E, Marion-Poll A (2003). ABA action and interactions in seeds. Trends Plant Sci.

[CR42] Ney B, Duthion C, Fontaine E (1993). Timing of reproductive abortions in relation to cell division, water content, and growth of pea seeds. Crop Sci.

[CR43] North HM, Almeida AD, Boutin J-P, Frey A, To A, Botran L, Sotta B, Marion-Poll A (2007). The *Arabidopsis* ABA-deficient mutant *aba4* demonstrates that the major route for stress-induced ABA accumulation is via neoxanthin isomers. Plant J.

[CR44] Ochatt SJ (2015). Agroecological impact of an *in vitro* biotechnology approach of embryo development and seed filling in legumes. Agron Sustain Dev.

[CR45] Ochatt S, Abirached-Darmency M, de Bruin FJ (2019). The underlying processes governing seed size plasticity: impact of endoploidy on seed coat development and cell expansion in *Medicago truncatula*. The model legume *Medicago truncatula*.

[CR46] Paauw M, Koes R, Quattrocchio FM (2019). Alteration of flavonoid pigmentation patterns during domestication of food crops. J Exp Bot.

[CR47] Ranathunge K, Shao S, Qutob D, Gijzen M, Peterson CA, Bernards MA (2010). Properties of the soybean seed coat cuticle change during development. Planta.

[CR48] Reinecke DM, Wickramarathna AD, Ozga JA, Kurepin LV, Jin AL, Good AG, Pharis RP (2013). Gibberellin 3-oxidase gene expression patterns influence gibberellin biosynthesis, growth, and development in pea. Plant Physiol.

[CR49] Ribalta FM, Pazos-Navarro M, Nelson K, Edwards K, Ross JJ, Bennett RG, Munday C, Erskine W, Ochatt SJ, Croser JS (2017). Precocious floral initiation and identification of exact timing of embryo physiological maturity facilitate germination of immature seeds to truncate the lifecycle of pea. Plant Growth Regul.

[CR50] Ribalta FM, Pazos-Navarro M, Edwards K, Ross JJ, Croser JS, Ochatt SJ (2019). Expression patterns of key hormones related to pea (*Pisum sativum* L.) embryo physiological maturity shift in response to accelerated growth conditions. Front Plant Sci.

[CR51] Rittenberg D, Foster GL (1940). A new procedure for quantitative analysis by isotope dilution, with application to the determination of amino acids and fatty acids. J Biol Chem.

[CR52] Schwartz SH, Zeevaart JAD, Davies PJ (2010). Abscisic acid biosynthesis and metabolism. Plant hormones: biosynthesis, signal transduction, action!.

[CR53] Siegel S, Castellan NJ (1988). Non parametric statistics for the behavioural sciences.

[CR54] Smýkal P, Vernoud V, Blair MW, Soukup A, Thompson RD (2014). The role of the testa during development and in establishment of dormancy of the legume seed. Front Plant Sci.

[CR55] Smýkal P, Nelson MN, Berger JD, Von Wettberg EJB (2018). The Impact of genetic changes during crop domestication. Agronomy.

[CR56] Sponsel VM (1983). The localization, metabolism and biological activity of gibberellins in maturing and germinating seeds of *Pisum sativum* cv. Progress No. 9. Planta.

[CR59] Tarkowská D, Strnad M (2018). Isoprenoid-derived plant signaling molecules: biosynthesis and biological importance. Planta.

[CR60] Turečková V, Novák O, Strnad M (2009). Profiling ABA metabolites in *Nicotiana tabacum* L. leaves by ultra-performance liquid chromatography–electrospray tandem mass spectrometry. Talanta.

[CR61] Urbanová T, Tarkowská D, Novák O, Hedden P, Strnad M (2013). Analysis of gibberellins as free acids by ultra performance liquid chromatography–tandem mass spectrometry. Talanta.

[CR62] Van Dongen JT, Ammerlaan AM, Wouterlood M, Van Aelst AC, Borstlap AC (2003). Structure of the developing pea seed coat and the post-phloem transport pathway of nutrients. Ann Bot.

[CR63] Wan L, Li B, Pandey MK, Wu Y, Lei Y, Yan L, Dai X, Jiang H, Zhang J, Wei G, Varshney RK, Liao B (2016). Transcriptome analysis of a new peanut seed coat mutant for the physiological regulatory mechanism involved in seed coat cracking and pigmentation. Front Plant Sci.

[CR64] Wang M, Li W, Fang C, Xu F, Liu Y, Wang Z, Yang R, Zhang M, Liu S, Lu S, Lin T, Tang J, Wang Y, Wang H, Lin H, Zhu B, Chen M, Kong F, Liu B, Zeng D, Jackson SA, Chu C, Tian Z (2018). Parallel selection on a dormancy gene during domestication of crops from multiple families. Nat Genet.

[CR65] Weber H, Borisjuk L, Wobus U (2005). Molecular physiology of legume seed development. Annu Rev Plant Biol.

[CR66] Xie X-B, Li S, Zhang R-F, Zhao J, Chen Y-C, Zhao Q, Yao Y-X, You C-X, Zhang X-S, Hao Y-J (2012). The bHLH transcription factor MdbHLH3 promotes anthocyanin accumulation and fruit colouration in response to low temperature in apples. Plant Cell Environ.

[CR67] Yamaguchi S (2008). Gibberellin metabolism and its regulation. Annu Rev Plant Biol.

[CR68] Zablatzká L, Balarynová J, Klčová B, Kopecký P, Smýkal P (2021). Anatomy and histochemistry of seed coat development of wild (*Pisum sativum subsp. elatius* (M. Bieb.) Asch. et Graebn. and domesticated pea (*Pisum sativum subsp. sativum* L.). Int J Mol Sci.

[CR69] Zhao L, Li M, Ma X, Luo D, Zhou Q, Liu W, Liu Z (2022). Transcriptome analysis and identification of abscisic acid and gibberellin-related genes during seed development of alfalfa (*Medicago sativa* L.). BMC Genom.

[CR70] Zhou R, Cutler AJ, Ambrose SJ, Galka MM, Nelson KM, Squires TM, Loewen MK, Jadhav AS, Ross ARS, Taylor DC, Abrams SR (2004). A new abscisic acid catabolic pathway. Plant Physiol.

[CR71] Zhu YX, Davies PJ, Halinska A (1991). Metabolism of gibberellin A12 and A12-aldehyde in developing seeds of *Pisum sativum* L. Plant Physiol.

