# Supplementary material for: Domestication has altered the ABA and gibberellin profiles in developing pea seeds
Source: Planta. 2023 Jun 23;258(2):25. doi: 10.1007/s00425-023-04184-2 (PMC10290032; doi:10.1007/s00425-023-04184-2)

**Supplementary Information**

**Supplementary Fig. S1** Arrangement of seeds (Cameor) in water loss experiment.

**
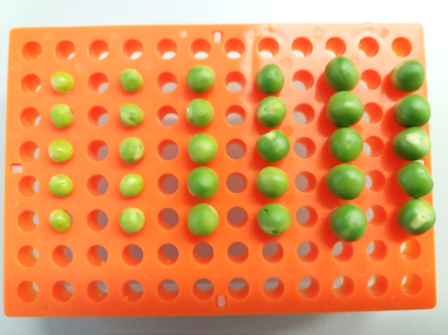
**

**Supplementary Fig. S2**The dry mature seeds of Cameor (**a**), JI92 (**b**) and JI1794 (**c**). The images were captured using the digital microscope VHX-7000 (Keyence). Scale bar = 2 mm (**a**) or 1mm (**b**, **c**).

**
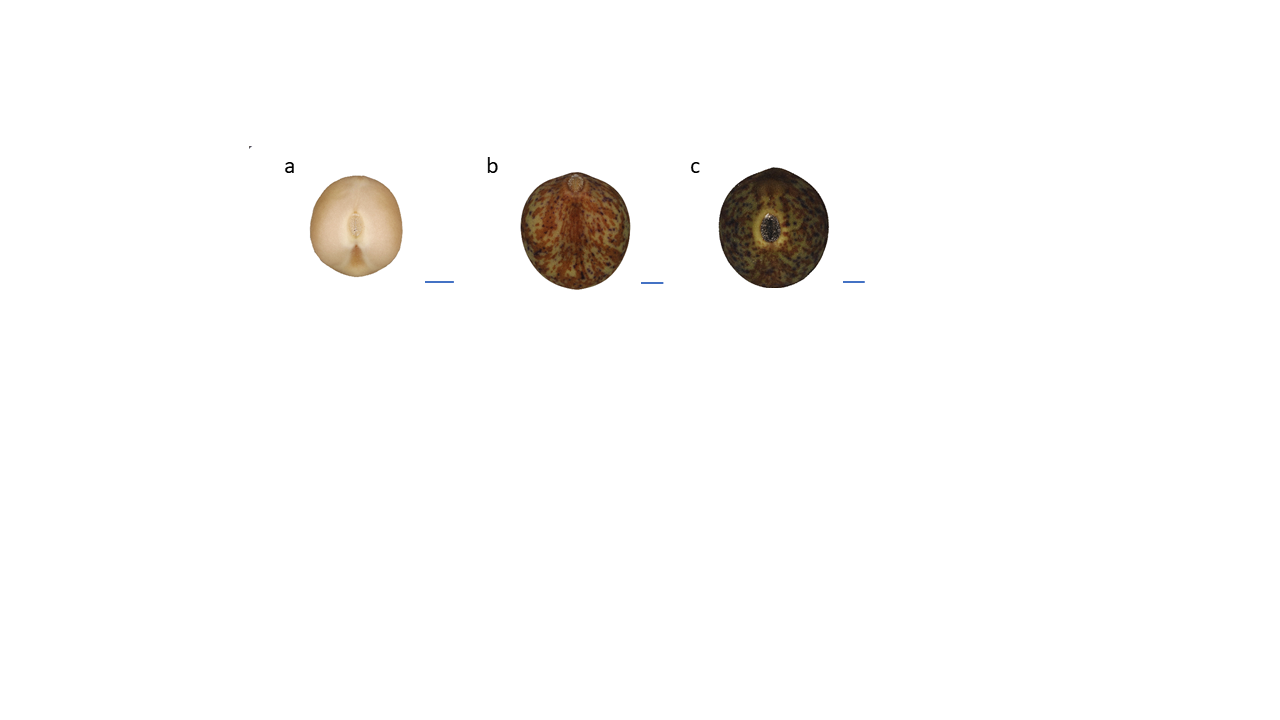
**

**Supplementary Fig. S3** Summary of statistical analysis of water loss during the development of Cameor, JI92 and JI64 seeds shown in Fig. 9. Different letters indicate significant differences (*P* = 0.05) by Kruskal-Wallis test with the following non-parametric multiple comparison test.


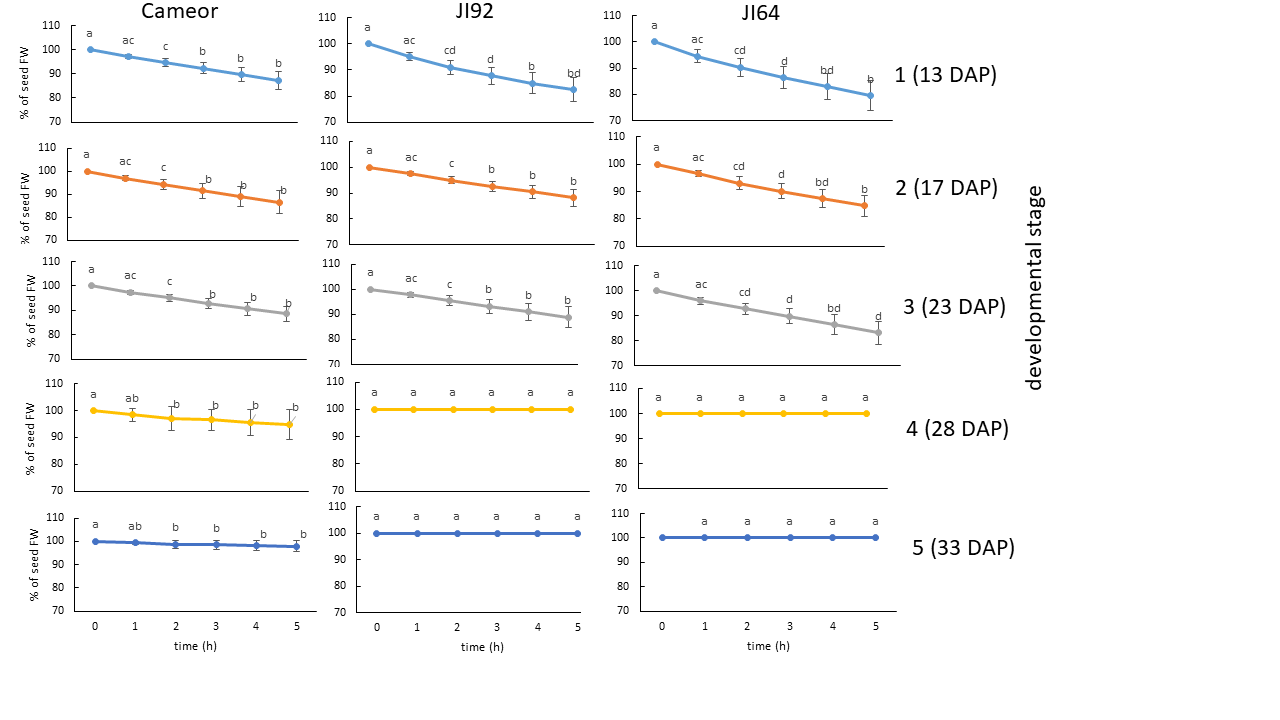

Supplement: Supplementary file 1 — Supplementary file1 (DOCX 620 KB) [file 425_2023_4184_MOESM1_ESM.docx]
