# Supplementary material for: Domestication has altered the ABA and gibberellin profiles in developing pea seeds
Source: Planta. 2023 Jun 23;258(2):25. doi: 10.1007/s00425-023-04184-2 (PMC10290032; doi:10.1007/s00425-023-04184-2)
Supplement: Supplementary file 2 — Suppl. File S1 Summary of statistical analysis of differences among genotypes. Different letters indicate significant difference (P = 0.05) by Kruskal–Wallis test with the following non-parametric multiple comparison test. Supplementary file2 (DOCX 43 KB) [file 425_2023_4184_MOESM2_ESM.docx]

Summary of statistical analysis of ABA levels in seed coats (SC) and embryos (E) of cultivated (Cameor and JI92) and wild (JI1794) pea genotypes during the seed development (stages 1, 2, 3 and 4) shown in Fig. 1. Different letters indicate significant difference (p = 0.05) between genotypes by Kruskal-Wallis test with the following non-parametric multiple comparison test.

Summary of statistical analysis of ABA glycosyl ester (ABA-GE), phaseic acid (PA), dihydrophaseic acid (DPA), 7'-hydroxy-ABA (7´-OH-ABA) and neophaseic acid (neoPA) levels in the seed coats (SC) and embryos (E) of cultivated (Cameor and JI92) and wild (JI1794) pea genotypes during the seed development (stages 1, 2, 3 and 4) shown in Fig. 2. Different letters indicate significant differences (p = 0.05) among genotypes in each developmental stage by Kruskal-Wallis test with the following non-parametric multiple comparison test.

Summary of statistical analysis of **the level of GA_1_** in the seed coats (SC) and embryos (E) of cultivated (Cameor and JI92) and wild (JI1794) pea genotypes during the seed development (stages 1, 2, 3 and 4) shown in Fig. 4. Different letters indicate significant differences (p = 0.05) among genotypes in each developmental stage by Kruskal-Wallis test with the following non-parametric multiple comparison test.

Summary of statistical analysis of **the level of GAs belonging to the 13-hydroxylation pathway** in the seed coats (SC) and embryos (E) of cultivated (Cameor and JI92) and wild (JI1794) pea genotypes during the seed development (stages 1, 2, 3 and 4) shown in Fig. 6. Different letters indicate significant differences (p = 0.05) among genotypes in each developmental stage by Kruskal-Wallis test with the following non-parametric multiple comparison test.

Summary of statistical analysis of changes in seed water content (WC) and dry weight (DW) during development (stages 1, 2, 3, 4 and 5) of cultivated (Cameor and JI92) and wild (JI64) pea seeds shown in Fig. 8. Different letters indicate significant differences (p = 0.05) among genotypes in each developmental stage by Kruskal-Wallis test with the following non-parametric multiple comparison test.

Summary of statistical analysis of water loss during development (stages 1, 2, 3, 4 and 5) of cultivated (Cameor and JI92) and wild (JI64) pea seeds shown in Fig. 9. Different letters indicate significant differences (p = 0.05) among genotypes by Kruskal-Wallis test with the following non-parametric multiple comparison test.
